# Resolution and dose dependence of radiation damage in biomolecular systems

**DOI:** 10.1107/S2052252519008777

**Published:** 2019-09-18

**Authors:** Hakan Atakisi, Lauren Conger, David W. Moreau, Robert E. Thorne

**Affiliations:** a Physics Department, Cornell University, Ithaca, NY 14853, USA; b Cornell University, Ithaca, NY 14853, USA

**Keywords:** protein crystallography, radiation damage, resolution, X-ray imaging

## Abstract

The local Fourier-space relation between diffracted intensity *I*, diffraction wavevector *q* and dose *D*, , is key to probing and understanding radiation damage by X-rays and energetic particles in both diffraction and imaging experiments. The models used in protein crystallography for the last 50 years provide good fits to experimental *I*(*q*) versus nominal dose data, but have unclear physical significance. More recently, a fit to diffraction and imaging experiments suggested that the maximum tolerable dose varies as *q* ^−1^ or linearly with resolution. Here, it is shown that crystallographic data have been strongly perturbed by the effects of spatially nonuniform crystal irradiation and diffraction during data collection. Reanalysis shows that these data are consistent with a purely exponential local dose dependence,  = *I*_0_(*q*)exp[−*D*/*D*_e_(*q*)], where *D*_e_(*q*) ∝ *q* ^α^ with α ≃ 1.7. A physics-based model for radiation damage, in which damage events occurring at random locations within a sample each cause energy deposition and blurring of the electron density within a small volume, predicts this exponential variation with dose for all *q* values and a decay exponent α ≃ 2 in two and three dimensions, roughly consistent with both diffraction and imaging experiments over more than two orders of magnitude in resolution. The *B*-factor model used to account for radiation damage in crystallographic scaling programs is consistent with α = 2, but may not accurately capture the dose dependencies of structure factors under typical nonuniform illumination conditions. The strong *q* dependence of radiation-induced diffraction decays implies that the previously proposed 20–30 MGy dose limit for protein crystallography should be replaced by a resolution-dependent dose limit that, for atomic resolution data sets, will be much smaller. The results suggest that the physics underlying basic experimental trends in radiation damage at *T* ≃ 100 K is straightforward and universal. Deviations of the local *I*(*q*, *D*) from strictly exponential behavior may provide mechanistic insights, especially into the radiation-damage processes responsible for the greatly increased radiation sensitivity observed at *T* ≃ 300 K.

## Abstract 2

Data from several radiation-damage studies using protein crystals are reanalyzed to determine the local dose- and resolution-dependent decay of diffracted intensity at *T* = 100 K. The results are inconsistent with long-accepted models and with a proposed linear scaling between maximum tolerable dose and resolution, but are reproduced by a simple, physics-based model.

## 1. Introduction   

Radiation damage is a key issue in all diffraction and imaging methods that illuminate biological samples with energetic particles such as X-ray photons, electrons, neutrons and positrons. Absorption and inelastic scattering processes transfer energy to the sample [quantified as dose *D*, the energy deposited per unit sample mass, in grays (Gy), where 1 Gy = 1 J kg^−1^], with initial energy deposition from each scattering event confined to a small volume that depends on the energy transfer (Nave & Hill, 2005 ▸; Sanishvili *et al.*, 2011 ▸; Finfrock *et al.*, 2013 ▸). Energetic electrons and reactive atomic and molecular species are generated, and diffuse and react, causing additional chemical, bond-scale damage (Holton, 2009 ▸; Garman, 2010 ▸). Accumulation of bond-scale damage causes degradation of sample order on larger and larger length scales (Warkentin *et al.*, 2013 ▸). In the absence of macroscopically disruptive damage processes such as the eruption of hydrogen bubbles (Massover, 2007 ▸; Meents *et al.*, 2009 ▸) (for example at very large dose rates; Warkentin *et al.*, 2017 ▸) or fracturing, this accumulation of damage manifests in imaging as a loss of image contrast or ‘blurring’, evident first in short length-scale features and then moving to larger and larger scales. In diffraction, damage manifests as a decrease in the diffracted intensity at large angles 2θ or large diffraction wavevectors *q* that progresses to smaller and smaller *q*. In addition to these ‘global’ effects of radiation damage, metal sites, disulfide bonds and other structures within the sample may be particularly sensitive to damage, giving rise to ‘site-specific’ damage (Ravelli & McSweeney, 2000 ▸; Weik *et al.*, 2000 ▸; Burmeister, 2000 ▸; Banumathi *et al.*, 2004 ▸).

In biomolecular crystallography, radiation damage limits the amount of diffraction data that can be collected per unit sample volume and introduces errors in experimental structure factors. The effects of radiation damage on individual Bragg peak intensities can be complex (Warkentin *et al.*, 2017 ▸). Aside from global *q*-dependent intensity decay and differential decays associated with site-specific damage, measured Bragg peak intensities depend on radiation-damage-induced broadening of crystal mosaicity and strain distributions, which can cause partially illuminated reflections to initially brighten with dose (Warkentin *et al.*, 2017 ▸). They also depend on the spatial pattern of sample irradiation during data collection (Bury *et al.*, 2018 ▸), which can lead to heterogeneous sample dose states within the illuminated volume and to measured diffraction intensities that reflect a complex convolution of spatially (and, at high dose rates, temporally) nonuniform damage effects (Warkentin *et al.*, 2017 ▸). Current crystallo­graphic processing software (Evans, 2006 ▸; Otwinowski *et al.*, 2012 ▸; Diederichs *et al.*, 2003 ▸; Diederichs, 2006 ▸) incompletely accounts for these effects. Refined structural models based on data with typical maximum resolutions of ∼2 Å are at least somewhat insensitive to these experimental errors, as models obtained from nominally zero-dose XFEL data sets show good general agreement with those from finite-dose synchrotron data sets (see, for example, Hirata *et al.*, 2014 ▸; Keedy *et al.*, 2015 ▸).

The most commonly used metric for characterizing the radiation sensitivity of biomolecular crystals is the half-dose *D*_1/2_, which is equal to the dose at which the integrated intensity within all observed diffraction peaks drops to half of its zero-dose value. Typical reported half-dose values for protein crystals are ∼10–30 MGy at 100 K and 100–400 kGy at room temperature (Leal *et al.*, 2013 ▸). Half-doses depend on the initial diffraction resolution of the crystal (Howells *et al.*, 2009 ▸), which depends on the crystal quality and size, the X-ray beam size and the amount of background scatter from air and surrounding liquid. Experimental half-doses also depend on the spatial distribution of dose within the crystal, which depends on the beam intensity profile, on the size and shape of the crystal and on whether the crystal is fixed or rotated during data collection (Warkentin *et al.*, 2017 ▸; Bury *et al.*, 2018 ▸), and on how dose is defined when irradiation is spatially non­uniform (Warkentin *et al.*, 2017 ▸). Furthermore, the way in which the half-dose weights the contribution of diffraction intensities at different *q* values is not obviously related to their information content or to their utility in defining and constraining the final structural model. Consequently, half-doses are at best a crude metric of radiation sensitivity and provide at best a rough rule-of-thumb limit in crystallographic data collection. Other metrics of radiation sensitivity such as the change in scaling *B* factor (Kmetko *et al.*, 2006 ▸; Leal *et al.*, 2013 ▸), scale (Leal *et al.*, 2013 ▸) and decay *R* factor (Diederichs, 2006 ▸) are used, but have related limitations.

Radiation damage has been more fully characterized using the *q*-dependent integrated intensity *I*(*q*), which is obtained by integrating sample diffraction within a wavevector shell of width Δ*q*, with values of diffraction resolution *d* = λ/2sin(θ) ∝ 1/*q* rather than *q* typically quoted (Sliz *et al.*, 2003 ▸; Bourenkov & Popov, 2010 ▸; Liebschner *et al.*, 2015 ▸). Plots of experimental *I*(*q*) for a given *q* (or resolution) shell versus time, time-integrated flux density (in photons cm^−2^) or nominal dose typically show an initial linear or exponential decay and then a more gradual decay at larger times or doses, giving an overall ‘hockey-stick’ shape when plotted on semi-log axes (for example as in Blake & Phillips, 1962 ▸; Warkentin *et al.*, 2017 ▸). The rate of the initial intensity decay with dose increases with increasing *q* (increasing resolution, corresponding to a decreasing numerical value of *d*). Kinetics-inspired models (Blake & Phillips, 1962 ▸; Hendrickson, 1976 ▸; Sygusch & Allaire, 1988 ▸) have been used for nearly 50 years to obtain good fits to measured *I*(*q*) versus exposure time or nominal dose, reproducing both the overall ‘hockey-stick’ shape at fixed *q* and its *q* or resolution dependence. However, the physical significance of the models and the fit parameters obtained have been unclear.

Recent experiments and analyses have emphasized the profound effects of nonuniform crystal irradiation during data collection on measured integrated intensity–dose curves (Warkentin *et al.*, 2017 ▸). For thaumatin and lysozyme crystals held in a fixed orientation and illuminated using an X-ray beam with a Gaussian intensity profile, the measured integrated (over all *q*) intensity versus time, time-integrated flux density or nominal dose has the ‘hockey-stick’ shape. Simulations show that the Gaussian illumination profile generates this shape even if the underlying relation between diffracted intensity and dose is strictly exponential. Since previous radiation-damage experiments have seldom, if ever, provided perfectly uniform crystal irradiation (even when the X-ray beams had nominally ‘top-hat’ profiles), interpretation of their intensity–dose curves using kinetics-inspired models is now suspect.

Our goal here is to determine the underlying ‘local’ Fourier-space relationship between damage and dose, 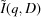, as it would be measured from a uniformly illuminated crystal. This relationship plays an analogous role in radiation-damage studies to the local relation between conductivity and electric field, σ(*E*), of conducting materials: it is the key to detailed understanding of damage and its mechanisms. Beginning with previously published experimental data at *T* = 100 K, we account for spatially nonuniform illumination during data collection and use this to estimate 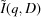. We then consider a simple model for radiation damage involving random, local disordering interactions. This model predicts a purely exponential 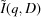 relation, and a decay constant that scales with *q* in a manner roughly consistent with experiment. Fits of previous models (Blake & Phillips, 1962 ▸; Hendrickson, 1976 ▸; Sygusch & Allaire, 1988 ▸) to experimental intensity versus resolution and dose data are invalidated as the primary trends have a different physical origin to that assumed. Experimental deviations from the predictions of the present model should illuminate how damage mechanisms evolve between cryogenic and room or biological temperatures.

## 2. Materials and methods   

### 2.1. Modeling and fitting experimental data for intensity versus dose and resolution   

In an ideal experiment to measure the local 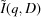 relation in a bulk crystal [where photoelectron escape from the crystal (Nave & Hill, 2005 ▸; Finfrock *et al.*, 2010 ▸; Sanishvili *et al.*, 2011 ▸) can be neglected], the entire crystal volume is illuminated with a fixed and uniform flux-density X-ray beam, and the crystal thickness along the incident beam direction is small compared with the X-ray absorption length. Under these illumination conditions, every crystal region receives the same dose *D* regardless of whether the crystal is rotated or held in a fixed orientation.

**Figure 1:**
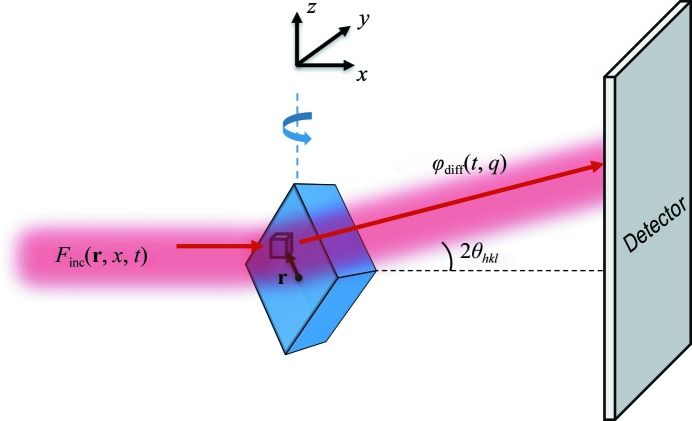
Experiment and simulation setup. A crystal (simulated as a rectangular prism) is located at *x*, *y*, *z* = 0 and a volume element (voxel) of the crystal is located at position **r**. The crystal is illuminated by an X-ray beam with a top-hat or Gaussian profile, and the incident photon flux at the voxel position is *F*_inc_(**r**, *x*, *t*). Crystal planes with spacing *d_hkl_* generate Bragg scattering at angle 2θ_*hkl*_, corresponding to a scattering wavevector *q_hkl_* = 2π/*d_hkl_*. The scattered flux (in photons s^−1^) from each voxel is φ_diff_(*t*, *q*).

In actual experiments (Fig. 1 ▸), the X-ray beam may be smaller than the crystal, the incident flux density within the beam may be nonuniform (even when nominally flat or ‘top-hat’ beams are used) and the crystal may be rotated during data collection. These result in different regions of the crystal experiencing different (and time-dependent) incident photon flux densities and dose rates, and accumulating different total doses *D*(*x*, *y*, *z*, *t*) (Diederichs, 2006 ▸; Bury *et al.*, 2018 ▸). This leads to spatially nonuniform damage. The measured diffracted intensity at any time is determined by both the incident X-ray flux-density distribution and by the distribution of damage states within the X-ray-illuminated sample volume (Diederichs, 2006 ▸; Warkentin *et al.*, 2017 ▸) [which can be characterized using an incident flux-density-weighted dose (Zeldin *et al.*, 2013 ▸) or, more meaningfully, by a diffraction-weighted dose (Warkentin *et al.*, 2017 ▸)]. Because the local relation between diffracted intensity and dose is in general nonlinear, knowing only the measured *I*(*q*) versus exposure time or nominal fluence (in photons mm^−2^) or nominal dose and the spatiotemporal pattern of crystal irradiation during data collection is not sufficient to uniquely determine the local 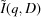 relation.

Thus, to analyze previous experimental *I*(*q*) data, we (i) calculate the spatial distribution of sample irradiation and dose from given experimental details, (ii) define a (somewhat) general expression for the local 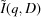 relation, with adjustable parameters, (iii) calculate the diffracted intensity *I*(*q*) versus nominal fluence or dose using this relation and the calculated dose distribution within the sample, and (iv) refine the parameters to optimize the quality of the fit to the *I*(*q*) data.

### 2.2. Calculating experimental dose distributions   

To calculate the diffracted flux from a crystal after a given exposure time, we first need to know the X-ray beam size and flux-density profile, the crystal size, shape, initial orientation and location in the X-ray beam, and how the crystal is rotated during the exposure. Using this information, we can calculate the total dose (in J kg^−1^) delivered to each volume element (voxel) of the crystal after time *t*: *D*(**r**, *t*). This dose determines the damage state of the voxel and the diffracted flux (in photons s^−1^) that it will produce per unit incident flux density. The diffracted flux from the crystal at time *t* is then obtained by summing the product of the incident flux density at each voxel and the diffracted flux per unit flux density of that voxel.

As shown in Fig. 1 ▸, a crystal of arbitrary shape is illuminated by an X-ray beam propagating along the *x* direction. During irradiation/data collection, the crystal may be rotated about an axis perpendicular to the beam direction, and we define a (stationary with respect to beam and detector) coordinate system oriented as shown with *x* = 0 located on the rotation axis and with *y* = *z* = 0 at the beam center. *F*_inc_[**r**(*t*), *x*, *t*] is the incident X-ray photon flux density (photons m^−2^ s^−1^), where **r** is a vector pointing from the coordinate system origin to the position of a voxel within the crystal at time *t*. The incident X-ray flux density decreases owing to scattering and absorption as it propagates through the crystal, and so in general is a function of the position *x* along the propagation direction.

Each voxel within the crystal is labeled with its initial (*t* = 0) Cartesian coordinates **r**_0_. As the crystal is rotated about the axis during data collection, a voxel at location **r**_0_ will rotate to location **r**(*t*) = **M**[φ(*t*)]**r**_0_ at time *t*, where **M** is a linear rotation matrix and φ(*t*) is the rotation angle about the axis. The dose that has been delivered at time *t* to a sample voxel initially located at **r**_0_ is given by
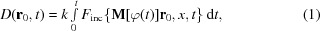
where the constant *k* depends on the X-ray energy and on the atomic composition and density of the crystal, and can be calculated using *RADDOSE*-3*D* (Bury *et al.*, 2018 ▸) or standard tables (Hubbell & Seltzer, 2004 ▸). We assume that the X-ray beam flux density is time-independent and that the crystal is thin compared with the X-ray attenuation length [a reasonable approximation when using 10–15 keV X-rays and 50–300 µm crystals; at 10 keV the beam is attenuated by an average of 17% on passing through 300 µm crystals of the proteins (lysozyme, thaumatin, apoferritin, HLA, λ3 and US2) of relevance here]. The incident flux density *F*_inc_(**r**, *x*, *t*) can then be written as *F*_inc_(ρ), where ρ = (*y* ^2^ + *z* ^2^)^1/2^ is the radial distance from the beam center.

### 2.3. Calculating diffracted intensities   

The crystal diffraction pattern measured on the detector consists of a large number of bright ‘spots’ corresponding to Bragg diffraction at angles (2θ)_*hkl*_ for which the scattering wavevector satisfies *q_hkl_* = 2π/*d_hkl_*, where *d_hkl_* is the spacing of a diffracting crystal lattice plane. Integrating the detector photon counts about the incident beam direction in radial bins of fixed width *dq* (variable width in detector coordinates) and dividing by the detector exposure time then gives a photon flux φ_diff_(*q*, *t*). At *t* = 0 the decay of φ_diff_(*q*, 0) with increasing *q* owing to thermal atomic motions and to static crystal disorder can be approximated by a Debye–Waller factor DWF(*B*, *q*).

At time *t* > 0, the diffraction at scattering wavevector *q* from each crystal voxel will be proportional to the incident flux density *F*_inc_(**r**, *x*, *t*) at the position of the voxel and to the diffracting power of the voxel (the number of diffracted photons per incident photon), which will depend on the dose received by the voxel from *t* = 0 to time *t*. Let Γ[*D*(**r**_0_, *t*), *q*] be the factor by which the diffracting power of a voxel at initial position **r**_0_ is reduced by radiation damage. The total diffracted flux from all crystal voxels at wavevector *q* per unit volume of reciprocal space, φ_diff_(*q*, *t*), is then
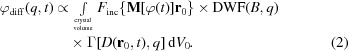



We assume that the diffracting power at a given *q* decays exponentially with dose,
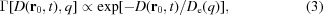
where *D*_e_(*q*) determines the decay rate. Experimentally, the scattered intensity decays more rapidly as *q* and 2θ increase, corresponding to a more rapid decay of short-wavelength Fourier components of the unit cell’s electron density. Based on a fit to experimental data from crystallography and X-ray imaging, Howells *et al.* (2009 ▸) suggested that *D*_e_(*q*) ∝ 1/*q* holds and that the resolution-dependent half-dose is given by *D*_1/2_(*d*) ≃ *d* × 10 MGy Å^−1^. We assume a more general relation
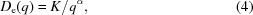
and then fit experimental data from crystallography to determine the exponent α.

The total diffracted photon flux of the crystal Φ_diff_ is obtained by integrating (3) over all *q* as
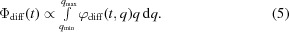



### 2.4. Fitting experimental data   

Fitting of reported data for diffracted intensity versus resolution *d* and nominal dose/fluence from prior experiments (Sliz *et al.*, 2003 ▸; Bourenkov & Popov, 2010 ▸; Liebschner *et al.*, 2015 ▸) was performed as follows, using the parameters given in Supplementary Tables S1 and S2.

We used either the actual reported beam profile (measured by scanning a slit across the beam) or else a profile matching the stated shape and width parameters (Supplementary Table S1). A ‘top-hat’ profile was represented as *F*_inc_(**r**) = *F*_0_ for ρ < ρ_max_ (or |*y*| < *y*_max_, |*z*| < *z*_max_) and 0 otherwise, and a Gaussian profile by 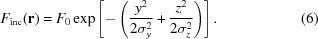



Each crystal was divided into cubic voxels, with roughly 100 voxels in each dimension. Crystals were assumed to be rectangular prisms with reported dimensions (Supplementary Table S1). Prisms were oriented as reported, and if no orientation was specified the orientation was adjusted to obtain the best fit to the intensity data (Section S1, supporting information). As a check, calculations were also performed assuming cylindrical crystals with their axes corresponding to the rotation axis.

The reported experiments either repeatedly oscillated the crystal through a small angle (*e.g.* 2°), collecting one or more frames for each oscillation and returning to the starting orientation before collecting the next set of frames, or else continuously rotated the crystal during data collection (*e.g.* by 60° with 1° rotation per diffraction frame). The former method gives a more uniform dose distribution in the irradiated and diffracting crystal regions. For simulations with repeated oscillations through an angular wedge, the simulation time step was set to 1/1000 of the total exposure time for the entire set of oscillations, the crystal was rotated 1/10 of its total oscillation in the wedge (*e.g.* by 0.5° for a 5° oscillation) in each step, and after every ten steps the rotation angle was reset to the starting angle of the wedge. For simulations with continuous rotations, the crystal was rotated by 1/100 of its maximum rotation in each simulation step. The diffracted flux versus *q*, φ_diff_(*t_n_*, *q*), the total diffracted flux φ_diff_(*t_n_*) and a nominal dose (an average over the voxels that have received nonzero dose) corresponding to the reported dose were calculated at each step. Supplementary Fig. S2 shows example dose distributions for a cylindrical crystal held in a fixed orientation and when rotated, when illuminated with flat-top and isotropic Gaussian beams.

Reported Bragg intensity data were integrated within resolution shells bounded by upper and lower *d* values. Reported incident beam profiles and diffracted intensity versus resolution shell and fluence/dose plots were digitized using the software *WebPlotDigitizer* (A. Rohatgi; https://automeris.io/WebPlotDigitizer/). Diffracted intensities in each shell were normalized by their zero-dose extrapolation, eliminating both the Debye–Waller factor DWF(*B*, *q*) in (2) as well as the Lorentz–polarization correction to the measured intensities, which was not consistently applied in all studies. The resulting normalized plots only show variations with resolution shell (*q*) and fluence or dose owing to radiation damage.

### 2.5. A simple physics-based model for radiation damage   

The primary model used to analyze global radiation damage to protein crystals for the last 50 years is due to Blake & Phillips (1962 ▸) and Hendrickson (1976 ▸), with an additional extension proposed later (Sygusch & Allaire, 1988 ▸). As shown in Supplementary Fig. S1, an undamaged crystal becomes dis­ordered at ‘rate’ *k*_1_ (proportional to the volume fraction of crystal disordered per unit dose). This damaged crystal continues to exhibit Bragg diffraction, but its intensities decrease with increasing *q* as *I*(*q*) = *I*_0_exp(−*B*_disorder_ *q* ^2^), where *B*_disorder_ is a (fixed) average *B*-factor increase in the disordered regions. Disordered crystal becomes completely amorphous and ceases to generate Bragg diffraction [*I*(*q*) = 0] at ‘rate’ *k*_2_. Undamaged crystal can also proceed directly to the amorphous state at rate *k*_3_. The resulting diffracted intensity versus *q* and dose *D* is given by
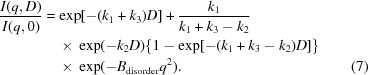
With four adjustable parameters, this model has provided good fits to reported *I*(*q*) versus nominal dose data, including to the ‘hockey-stick’ dose dependence evident for data at larger *q* or in higher (smaller numeric) resolution shells (Blake & Phillips, 1962 ▸; Hendrickson, 1976 ▸; Sliz *et al.*, 2003 ▸; Bourenkov & Popov, 2010 ▸; Warkentin & Thorne, 2010 ▸; Liebschner *et al.*, 2015 ▸; Owen *et al.*, 2014 ▸; Warkentin *et al.*, 2017 ▸). However, the connection of this model to underlying damage processes is opaque and the physical significance of the obtained fit parameters is unclear.

We thus considered a very simple model that captures some essential physical aspects of radiation damage. Incident X-ray photons interact with the sample at random times and locations. Each interaction ejects a photoelectron, which then generates lower energy secondary electrons within a volume (determined by the photoelectron mean free path) of a few micrometres. These secondary electrons then diffuse, break bonds and create free radicals that can diffuse and cause additional damage. Experiments using X-ray microbeams (Sanishvili *et al.*, 2011 ▸; Finfrock *et al.*, 2013 ▸) show that the resulting perturbations to electron densities from those of the original, undamaged crystal, as reflected in the degradation of the diffraction properties, are confined to within a length comparable in magnitude to the photoelectron mean free path, even at room temperature (Warkentin *et al.*, 2017 ▸) where diffusion occurs freely.

To model damage, we thus assume that X-ray photon interactions occur at random locations in the crystal. We model the effect of each interaction as a modest ‘blurring’ of the real-space electron density within a finite region around each interaction point. The number of such interactions per unit crystal volume determines the dose *D*. We calculate the Fourier transform of the electron density of the crystal and evaluate the integrated Bragg intensity within *q* (or resolution) shells and plot this versus dose *D*. Simulations were performed in two dimensions, using *m* × *m* arrays of unit cells containing a grayscale image of a flea (Fig. 5), and in three dimensions, using unit cells obtained by discretizing the protein in PDB entry 3e4h (Wang *et al.*, 2009 ▸): tetragonal crystals of the 29-residue plant protein cyclotide varv F at 1.8 Å resolution (Supplementary Fig. S6).

In two dimensions, for each X-ray hit a Gaussian spatial filter was applied to a small *n* × *n* pixel interaction region centered at a randomly chosen crystal location (*x_i_*, *y_j_*) as shown in Supplementary Fig. S7. FFTs of the crystal were periodically calculated as hits accumulated. The simulations were continued until the random hits caused the diffraction peaks in the highest resolution shell to fall below the background level, which corresponded to roughly 5–10 hits per pixel. A similar procedure was followed for the computationally more intensive three-dimensional simulations. Details of the simulations are given in Section S2 of the supporting information.

## 3. Results   

### 3.1. Fitting experimental intensity versus dose data   

Experimental data for intensity in resolution shells versus dose at *T* ≃ 100 K from three previous studies were analyzed, modeled and fitted using the approach in Sections 2.1–2.5. Supplementary Tables S1 and S2 give the experimental details for each reference and our model parameters. We focused on cryogenic temperature data because crystals of different proteins are comparably radiation sensitive, damage is independent of dose rate and because free-radical diffusion and relaxation of protein and lattice structure following bond-scale damage are strongly constrained by the frozen solvent matrix, so that the overall behavior should be simpler and more consistent between protein crystals than at 300 K.

#### 3.1.1. Liebschner *et al.* (2015 ▸)   

**Figure 2:**
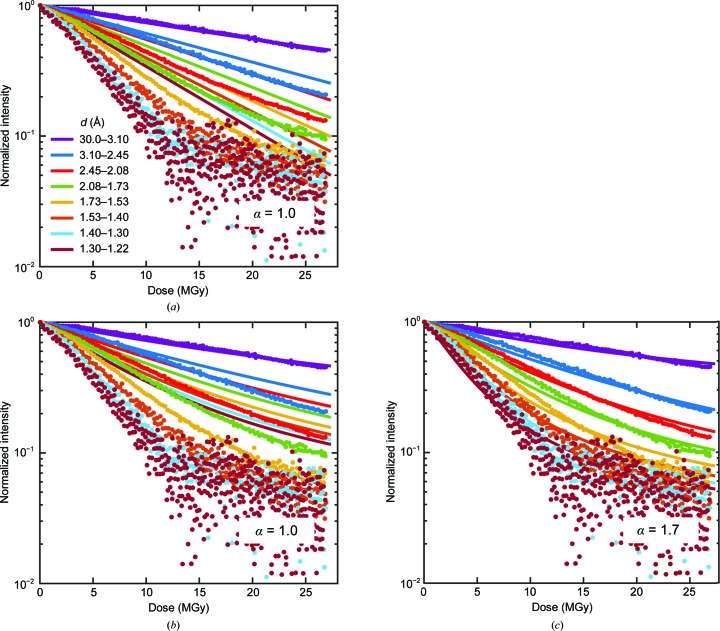
Experimental data (solid circles) for integrated intensity in resolution shells versus dose for thaumatin crystals at 100 K, as measured by Liebschner *et al.* (2015 ▸). Absolute intensities in each resolution shell (Fig. 4 in the original manuscript) have been normalized by the first (approximately zero-dose) intensity point; non-normalized data are shown in Supplementary Fig. S4. The solid lines indicate results from simulations assuming (*a*) a perfect top-hat incident X-ray beam profile and an exponent α = 1 in (4), (*b*) the measured beam profile (Fig. 1 in the original manuscript, reproduced as Supplementary Fig. S3) and α = 1, and (*c*) the measured beam profile and a best-fit exponent α = 1.7.

Liebschner *et al.* (2015 ▸) reported the most optimally executed experiments of those examined here. Data were collected from thaumatin crystals at 100 K by repeatedly oscillating the crystals through the same 2° range. Their measured beam profile (their Fig. 1, reproduced here as Supplementary Fig. S3) was nominally flat-topped but had significant tails, and the full widths at half maximum (FWHMs) were much smaller than the crystal dimensions. Fig. 2 ▸ shows their data for normalized integrated intensity in resolution shells versus nominal dose. As the resolution of the shell increases, the initial decay rate with dose becomes more rapid, and deviations above exponential behavior become evident at smaller doses.

The solid lines in Fig. 2 ▸(*a*) show the calculated intensities assuming a top-hat incident beam profile, a 2° oscillation and an exponent α = 1 for the *q* (resolution) dependence of the diffracted intensity decay with dose in (4). The calculated dose variation is nearly perfectly exponential and thus does not capture the large deviations from exponential behavior at higher resolutions and doses. By using the measured beam profile [Fig. 2 ▸(*b*)], the non-exponential behavior at higher resolutions is qualitatively reproduced. However, in both Figs. 2 ▸(*a*) and 2 ▸(*b*) the choice of α = 1, motivated by the results of Howells *et al.* (2009 ▸), seriously underestimates the observed increase in decay rate with increasing resolution. As shown in Fig. 2 ▸(*c*), relaxing this constraint yields a best-fit value of α ≃ 1.7 and agreement with the data that is generally excellent in all resolution shells.

#### 3.1.2. Sliz *et al.* (2003 ▸)   

Sliz *et al.* (2003 ▸) collected data at 100 K from crystals of three different proteins: the ternary US2–HLA-A2–Tax peptide complex (referred to as ‘US2’), HLA-A2 with a bound melanoma decamer peptide (referred to as ‘HLA’) and viral polymerase λ3 from reovirus (referred to as ‘λ3’). The focused and collimated X-ray beam was assumed to have a top-hat form in the collimated horizontal direction and a Gaussian form in the focused vertical direction. The crystals were oscillated by only 1°, and all were larger than the beam. Intensities were plotted versus incident fluence (photons mm^−2^, proportional to dose) and data at low and high fluences were separately reported.

**Figure 3:**
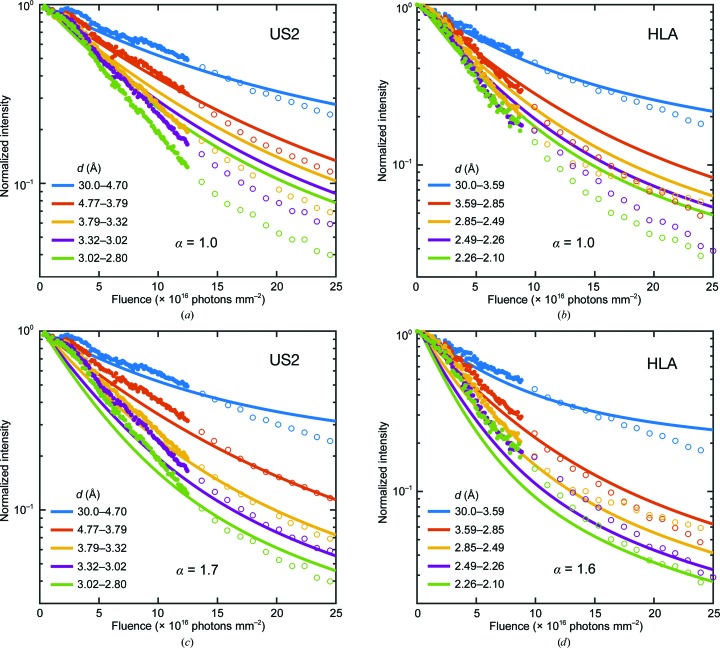
Experimental data in the low-dose (solid circles) and high-dose (open circles) regions for integrated intensity in resolution shells versus incident fluence (in photons mm^−2^, proportional to dose) reported by Sliz *et al.* (2003 ▸) (Fig. 1 in the original manuscript) for crystals of US2 and HLA at 100 K. The solid lines indicate results from simulations assuming a top-hat incident beam profile in the horizontal direction and a Gaussian profile in the vertical direction (based on descriptions of the experimental setup), with (*a*), (*b*) α = 1 and (*c*), (*d*) ‘best-fit’ values chosen based on visual comparison.

As shown in Fig. 3 ▸ and Supplementary Fig. S5, these data again show a faster increase in decay with resolution than can be accounted for with α = 1. Fit values were 1.7 for US2, 1.6 for HLA and 1.2 for λ3. Poorer fits at all resolutions and much larger uncertainties in ‘best-fit’ α values than for the data of Liebschner and coworkers result because of obvious problems with the original data, and because the beam profiles and initial crystal orientations were not reported. Intensities for all three proteins show an initial plateau (US2) or reduced slope (HLA and λ3) versus fluence. Similar behavior observed for thaumatin and lysozyme crystals has been attributed to the effects of dose-dependent mosaicity broadening and cell expansion (Warkentin *et al.*, 2017 ▸), which is not accounted for by scaling algorithms or by our modeling.

#### 3.1.3. Bourenkov & Popov (2010 ▸)   

**Figure 4:**
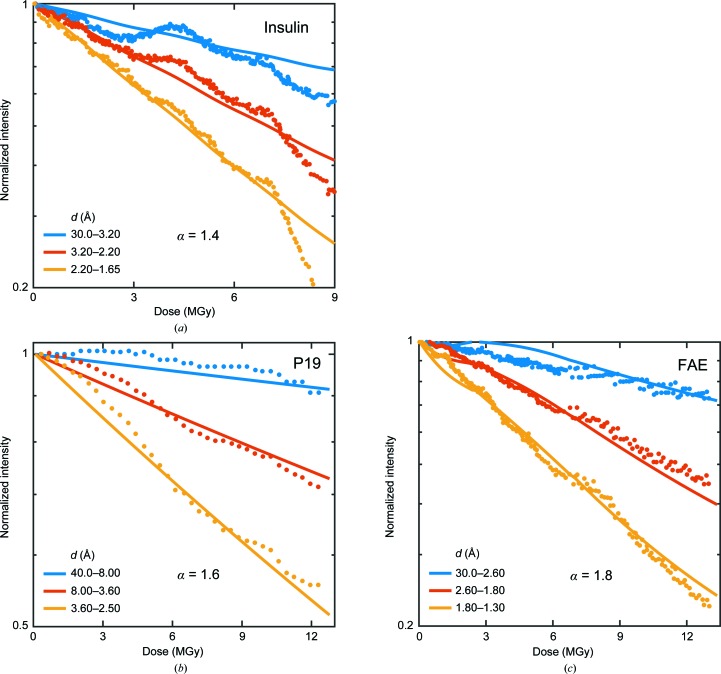
Experimental data (solid circles) for integrated intensity in resolution shells versus dose for crystals of insulin, P19 and FAE at 100 K, as measured by Bourenkov & Popov (2010 ▸) (Figs. 3, 4 and 6 in the original paper). Crystals were rotated continuously during irradiation as for crystallographic data collection. Oscillations in the data may be owing to irregular crystal shapes and large rotations that produce complex dose distributions within the X-ray-illuminated volume. The solid lines indicate the results from simulations with best-fit exponents α of (*a*) 1.4, (*b*) 1.6 and (*c*) 1.8.

Bourenkov & Popov (2010 ▸) collected data at 100 K from crystals of insulin, P19-siRNA, FAE and FtsH. Crystals of the first three were rotated during exposure by a total angle of between 35 and 300°, with 0.5–1° rotation per frame. Large rotations are not ideal for our modeling because the dose distribution within and diffraction from the illuminated volume will have a larger dependence on the detailed crystal shape and initial orientation than when crystals are oscillated through a small angle. Crystals of FtsH were both rotated and translated (after each 30°, by an unknown amount) perpendicular to the beam direction, and their data were not modeled. Crystals were illuminated by a nominally Gaussian beam with dimensions that were equal to or smaller than the largest crystal dimension. Beam profiles and initial crystal orientations were not reported. Despite many uncertainties the model calculations yield good fits to the data for all three crystals (Fig. 4 ▸), with best-fit exponents of α = 1.4, 1.6 and 1.8 for insulin, P19 and FAE, respectively.

### 3.2. Simulations of radiation damage   

**Figure 5:**
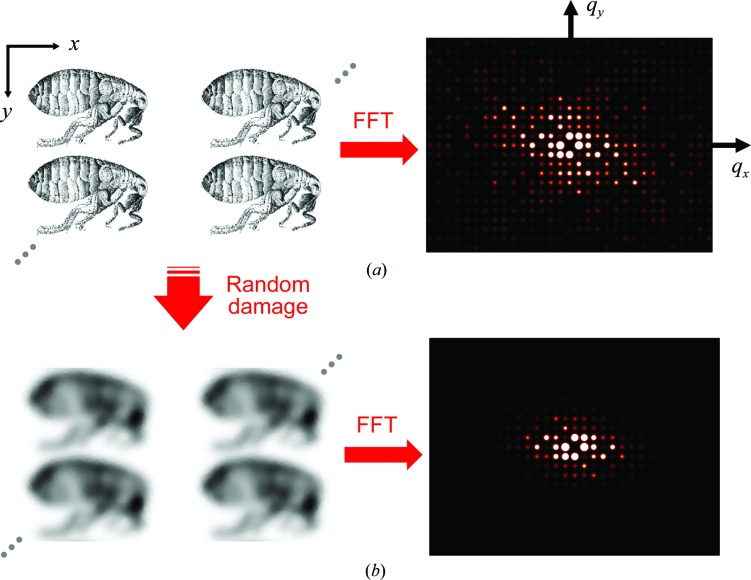
Simulation of radiation damage to a two-dimensional crystal formed of images of a flea (from Robert Hooke’s *Micrographia*). Each ‘hit’ corresponds to the application of a local Gaussian blur at a randomly selected location (Supplementary Fig. S7). (*a*) Four unit cells (each 1024 × 512 pixels) of the undamaged two-dimensional crystal (left) and the square of the FFT amplitude, proportional to the diffracted intensity, of a 16 × 16 cell crystal (right). (*b*) After receiving a large number of hits (>>1 per pixel) the electron density has been blurred and the high-resolution (large *q*) diffraction peaks have faded out. A full video of the evolution of the crystal and its diffraction pattern with dose is given in Supplementary Movies S1 and S2.

Figs. 5 ▸ and 6 ▸ show the results of simulations of our model for radiation damage as a sequence of random, local Gaussian blurs and corresponding to a condition of spatially uniform irradiation. Fig. 5 ▸(*a*) shows four unit cells of an initial, un­damaged 16 × 16 cell two-dimensional crystal. Its diffraction (proportional to the square of the FFT amplitudes) has strong peaks extending out to the maximum *q* or resolution of the initial image. After some large number of hits, the real-space density is blurred throughout the crystal and its diffraction decays much more rapidly with *q*. Movies of the evolution of the electron density of the crystal and its diffraction are provided as Supplementary Movies S1 and S2.

**Figure 6:**
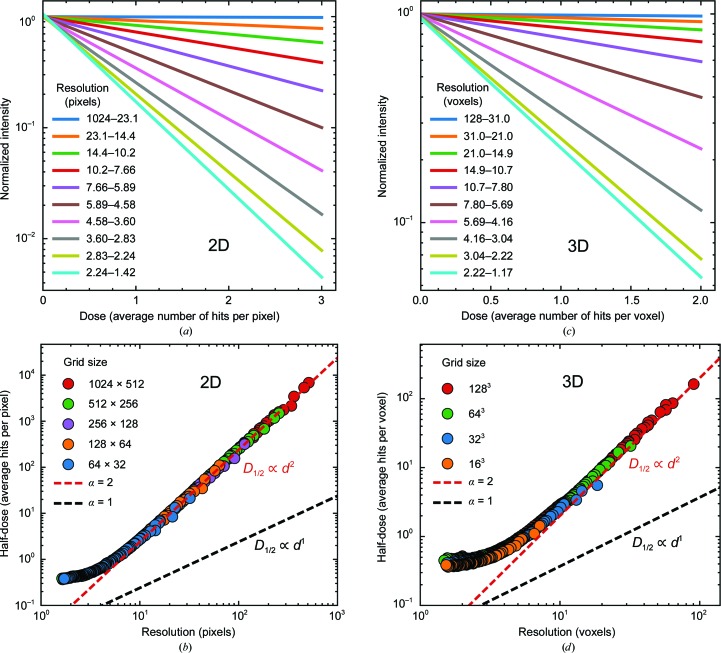
Simulation results for the random Gaussian blur model of radiation damage applied to (*a*, *b*) a two-dimensional crystal formed of pixelated images of a flea and (*c*, *d*) a three-dimensional crystal formed of a pixelated electron-density map for PDB entry 3e4h. In both two and three dimensions, the intensity within a given resolution shell decays exponentially with dose, and the half-dose varies with resolution *d* approximately as *D*_1/2_(*d*) ∝ *d* ^α^, with α approaching 2 at low resolutions (large numeric values of resolution). The best-fit values in the near-linear region are α = 1.95 and α = 1.86 in (*c*) and (*d*), respectively. Simulation details are given in the supporting information.

Figs. 6 ▸(*a*) and 6 ▸(*c*) give results in two and three dimensions, respectively, for the predicted diffracted intensity in a resolution shell versus dose. In both two and three dimensions, the diffracted intensity within a resolution shell has a strictly exponential decay with dose, consistent with our assumption for the behavior of the local 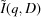 in fitting the data in Figs. 2 ▸, 3 ▸ and 4 ▸. Figs. 6 ▸(*b*) and 6 ▸(*d*) give results in two and three dimensions, respectively, for the half-dose in a given resolution shell versus resolution, determined from plots as in Figs. 6 ▸(*a*) and 6 ▸(*c*) as the dose at which the intensity in a given resolution shell drops to half of its initial value. Except at the highest (lowest numerical value) resolutions, in both two and three dimensions the half-dose varies with resolution *d* approximately as *D*_1/2_(*d*) ∝ *d* ^α^ with an apparent low-resolution asymptote of α ≃ 2; best-fit values to the near-linear regions in the two- and three-dimensional results are α ≃ 1.96 and 1.86, respectively. These results do not change when the Gaussian blur is replaced by a uniform blur (Supplementary Fig. S8). Deviations from simple power-law behavior at the highest (lowest numerical value) resolutions depend on the Gaussian width, with larger widths causing deviations at lower resolutions. Similar results were obtained using other two-dimensional images and using other PDB entries as the basis for the three-dimensional unit cell.

## 4. Discussion   

### 4.1. The local *I* ~(*q*, *D*) relation: dose dependence   

By assuming a purely exponential local dependence of diffracted intensity on dose of the form 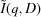 = *I*_0_(*q*)exp[−*D*/*D*_e_(*q*)] with *D*_e_(*q*) = *K*/*q* ^α^, and accounting for the nonuniform pattern of crystal irradiation during data collection, we obtain good fits to experimental *I*(*q*) versus nominal dose/fluence relations measured for several protein crystals at *T* = 100 K under diverse data-collection conditions. Deviations of the calculated dose/fluence dependence from the data may arise because the actual crystal shapes and initial crystal orientations (which were not given or adequately described) deviate from those assumed, and because of issues in data collection and processing that cause measured intensities to deviate from the actual dose-dependent structure factors (Warkentin *et al.*, 2017 ▸). Consequently, based on the available data at 100 K, there is no reason to believe that the local 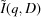 relation at *T* ≃ 100 K is anything but purely exponential in all resolution shells over the resolution and dose range relevant in biomolecular crystallography.

The present analysis also shows that even relatively small deviations of the profile of an X-ray beam from an ‘ideal’ top-hat form can have a substantial effect on the dose dependence of the intensity at larger doses and higher resolutions. This is particularly evident in the fits to the data of Liebschner and coworkers in Fig. 2 ▸; using the actual profile of the nominally top-hat beam dramatically improves the fit quality.

### 4.2. The local *I* ~(*q*, *D*) relation: *q* dependence   

Howells *et al.* (2009 ▸) presented a summary of available data for resolution-dependent maximum tolerable doses, obtained from published half-dose values in biomolecular crystallo­graphy and from X-ray and electron imaging studies. These results, spanning resolutions from ∼2 to 700 Å, show large scatter but are roughly consistent with a linear resolution dependence corresponding to *D*_e_(*q*) = *K*/*q* ^α^ with α = 1.

The present analysis shows that data for protein crystallo­graphy with resolutions between ∼1 and 10 Å are unambiguously inconsistent with α = 1, and yield best-fit values of between ∼1.4 and 1.8, with the most ‘ideal’ data of Liebschner and coworkers yielding a value of ∼1.7.

**Figure 7:**
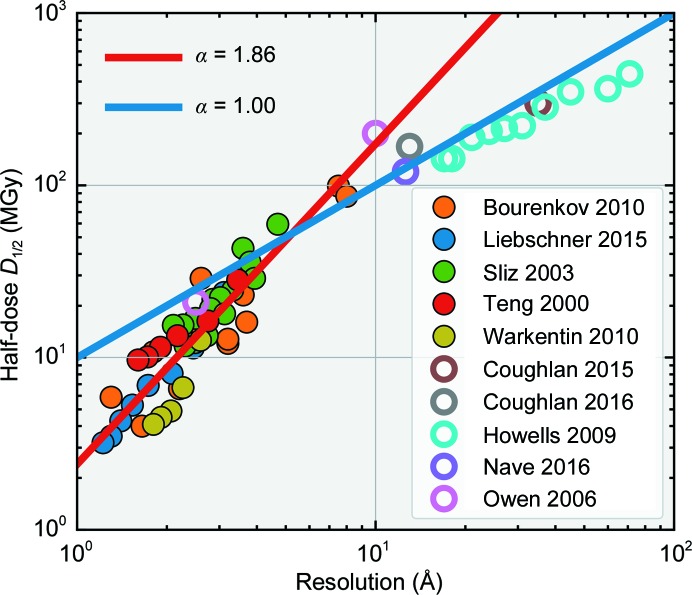
Experimental half-doses versus resolution obtained from several previous crystallographic studies (Bourenkov & Popov, 2010 ▸; Liebschner *et al.*, 2015 ▸; Sliz *et al.*, 2003 ▸; Teng & Moffat, 2000 ▸; Warkentin & Thorne, 2010 ▸; Coughlan *et al.*, 2015 ▸, 2016 ▸; Howells *et al.*, 2009 ▸; Nave *et al.*, 2016 ▸; Owen *et al.*, 2006 ▸), analyzed accounting for their dose distribution (solid circles) or used as reported (open circles). Aside from the data of Howells *et al.* (2009 ▸) at resolution beyond 10 Å, the overall trend is in good agreement with α = 2, with a best-fit exponent of 1.86.

Fig. 7 ▸ summarizes the results for half-dose versus resolution at 100 K, deduced from previous *I*(*q*) measurements using the methods of Sections 2.1–2.5 (solid symbols) or as originally reported (open symbols). These half-dose values are for diffraction within resolution shells, rather than overall half-dose values obtained by integrating the entire diffraction pattern over all resolutions. The data between 1 and 10 Å are well described by α ≃ 2, with a best-fit value of 1.86; the only data that appreciably deviate from this fit are those first reported by Howells and coworkers. Extrapolating the α = 1.86 fit from 1 to 600 Å yields a half-dose of ∼3 × 10^11^ Gy and using α = 2 gives ∼7 × 10^11^ Gy. This compares with a reported overall half-dose value (obtained by integrating over all resolutions) in X-ray imaging of cells to this resolution of 5 × 10^11^ Gy (Maser *et al.*, 2000 ▸) and with a value of only ∼1 × 10^10^ Gy based on the best fit in Howells and coworkers (their Fig. 3) with α = 1.

### 4.3. The local *I* ~(*q*, *D*) relation: connection to ‘kinetic’ models   

The ‘kinetic’ models of Blake & Phillips (1962 ▸), Hendrickson (1976 ▸) and Sygusch & Allaire (1988 ▸) all implicitly assume uniform sample irradiation, and so should not have been used to fit experimental data that were collected under conditions of substantially nonuniform illumination. The present analysis shows that the local 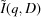 is consistent with a purely exponential dose dependence for all *q* at 100 K and that the dose scale for intensity at a given *q* varies as a power of *q*. Equation (7) cannot replicate these features with any sensible parameter choices. The deviations from exponential behavior that these models have proved so successful at fitting are owing to non­uniform sample irradiation (and possibly also to data-processing errors), which these models do not include. These models are thus inconsistent with experiment at *T* = 100 K and should no longer be used.

### 4.4. The ‘dose limit’ in biomolecular crystallography   

Based upon experience in cryoelectron microscopy, Henderson (1990 ▸) suggested that the maximum tolerable dose in X-ray cryocrystallography, beyond which diffraction would be seriously degraded, would be roughly 20 MGy (Henderson, 1990 ▸). Teng & Moffat (2000 ▸, 2002 ▸), using perhaps the most nearly ideal irradiation conditions to date – a beam with a 2σ width much larger than their crystal size (250 µm versus 100 µm), giving nearly uniform illumination of the entire crystal volume – obtained a *T* = 100 K half-dose of ∼17 MGy for lysozyme crystals diffracting to 1.6 Å resolution. Based on diffraction statistics they suggested a dose limit of ∼10 MGy be used in macromolecular crystallography. Bur­meister (2000 ▸) obtained a *T* = 100 K half-dose of ∼21 MGy for myrosinase crystals diffracting to 2.0 Å resolution. Owen *et al.* (2006 ▸) obtained half doses of 40 and 48 MGy for holoferritin and apoferritin crystals diffracting to ∼2.3 Å, and based on examination of diffraction statistics and electron-density maps suggested that a maximum dose of ∼30 MGy be used. Liebschner *et al.* (2015 ▸) using a nearly flat-top beam, obtained a half-dose of 18.5 MGy for thaumatin crystals diffracting to 2.1 Å. Warkentin *et al.* (2017 ▸), using a Gaussian microbeam with no crystal oscillation and correcting for the effects of the Gaussian beam profile, obtained half-doses of 10 MGy for lysozyme crystals diffracting to 1.4 Å and 13 MGy for thaumatin crystals diffracting to 1.6 Å. Many other half-dose measurements have been reported but have generally involved substantially non­uniform crystal irradiation, which can make measured half-doses substantially larger than the true, local half-dose that would be measured under conditions of purely uniform irradiation (Warkentin *et al.*, 2017 ▸).

Our conclusion based these previous studies is that diffraction half-doses at *T* = 100 K for crystals diffracting to ∼1.5–2 Å are ∼15–20 MGy, and that the ∼10 MGy dose limit suggested by Teng and Moffat is appropriate. However, the 30 MGy ‘Garman limit’ reported by Owen *et al.*, rather than the 10 MGy limit of Teng and Moffat, has been by far the most widely cited, and has become the accepted standard dose limit. It far exceeds the dose that would be required to severely degrade diffraction in the highest resolution shells of, for example, the overwhelming majority of PDB entries, which have a median refined resolution of ∼2.0 Å.

As shown in Fig. 7 ▸, using the *local* half-dose in a given resolution shell as a more meaningful and robust metric, we find that the dose limit at *T* = 100 K increases from ∼2–3 MGy at 1 Å to 8 MGy at 2 Å, 16 MGy at 3 Å and 30 MGy at 4 Å. Half-doses obtained by integrating over all resolutions up to the maximum available resolution are somewhat larger than but track these values; for lysozyme crystals diffracting to a maximum resolution of 1.4 Å the half-dose is ∼10 MGy (Warkentin *et al.*, 2017 ▸). These resolution-dependent dose limits should be used as rules of thumb in place of the previous 20 or 30 MGy limit when determining exposure strategies in crystallographic measurements.

Why were the half-doses reported by Owen *et al.* so much larger than were obtained in the other measurements? As noted previously (Warkentin *et al.*, 2014 ▸), the data sets analysed had a resolution limit of 2.3 Å, somewhat lower than those used in other studies; this can account for roughly half the difference with half-dose values measured by Teng and Moffat (Teng & Moffat, 2000) and Warkentin *et al.* (Warkentin *et al.*, 2017 ▸). Owen *et al.* used a 100 × 100 µm X-ray beam, and stated that the beam profile on the beamline used for their studies ‘has been determined to be a top-hat shape’, citing Arzt *et al.* (Arzt *et al.*, 2005 ▸) for the profile. However, the beamline profiles reported in Fig. 4 of Arzt *et al.*, are not top hat (normally produced through collimation or slitting of a defocused beam). They are standard focused profiles. With tight focusing to 31 (*V*) × 47 (*H*) µm [Arzt *et al.*, 2005 ▸; Fig. 4(*a*)], the beam profile was roughly Gaussian in the vertical and somewhat flattened in the horizontal; with a 100 × 100 µm FWHM spot size as used by Owen *et al.*, the profile may have been more nearly Gaussian in both horizontal and vertical [Arzt *et al.*, 2005 ▸; Fig. 4(*b*)]. Since the beam size was much smaller than the ∼200 µm of the holo- and apoferritin crystals examined, the crystals may thus have been nonuniformly irradiated, and this may have increased the apparent half-dose relative to the true, local half-dose by a factor close to two.

Which experimental dose should be compared with these limits, for a crystal diffracting to a given maximum resolution? In nearly all crystallographic data collection the crystal is non­uniformly irradiated owing to nonuniform flux density in the beam and owing to crystal rotation. Using *RADDOSE*-3*D* or simple code (written in, for example, *MATLAB*) based on (1), and knowing the incident X-ray flux-density profile, the approximate crystal dimensions and the crystal rotation or oscillation pattern, the dose distribution within a crystal during a given data collection can easily be calculated. *RADDOSE*-3*D* currently calculates the maximum dose (at any position) received within the X-ray-illuminated crystal volume, the average dose within the illuminated volume, the average dose within the crystal and an incident flux-density-weighted dose (Zeldin *et al.*, 2013 ▸). The most conservative choice is to use the maximum dose. The average dose within the illuminated volume and within the crystal can both yield problematically small dose estimates when the crystal is larger than the beam and when the beam has a non-top-hat (*e.g.* Gaussian) profile.

A more robust measure of average dose is the diffraction-weighted dose (Warkentin *et al.*, 2017 ▸), which weights the dose received at each location after an exposure time *t* by its contribution to the measured diffraction at time *t*, and thus appropriately downweights contributions from regions that, either owing to weak incident illumination or owing to radiation damage, contribute little to the measured diffraction. The diffraction-weighted dose is given by
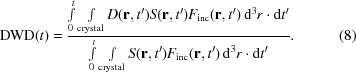
Here, *S*(**r**, *t*) = *S*[*D*(**r**, *t*)] is the diffracted flux (in all reflections) per unit illuminated crystal volume per unit incident flux density at position **r** and time *t*, and the denominator gives the total number of scattered photons up to time *t* (not the total diffracted intensity as stated by Warkentin and coworkers). As assumed by Warkentin and coworkers and supported by the present analysis, *S*(**r**, *t*) decays exponentially with dose *D*. However, since the highest resolution diffraction peaks fade out at the smallest doses, they are down-weighted in the definition of (8). If the effects of dose within a given resolution shell centered at some *q* are of primary interest, then the weighting diffraction can be restricted to that *q*, *e.g.*

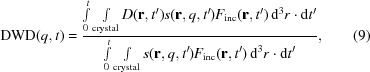
where 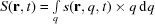
and, averaging over reflections at a given *q* as in our model in Section 2.3, *s*(**r**, *q*, *t*) ≡ Γ[*D*(**r**, *t*), *q*] × DWF(*B*, *q*).

### 4.5. Mechanisms underlying the local *I*(*q*, *D*) relation   

Global radiation damage to biomolecular crystals at 100 K is robust: it does not have an appreciable dependence on any properties of the biomolecule (*e.g.* primary sequence and fold) or of the crystal (*e.g.* packing density, solvent content and composition). Each X-ray absorption or inelastic scattering event generates secondary electrons and damage in a volume of many cubic micrometres containing a large number of biomolecules and unit cells. The frozen solvent network prevents relaxation of the structure following each damage event on any but the shortest length scales. As a result, one might expect that a fairly simple physical damage model would be required to reproduce the essential features of the decay of diffraction with dose at 100 K.

This expectation is borne out by the present results. A model of sequential random damage interactions that cause local blurring of the electron density reproduces all salient trends of the available data for global radiation damage, including both its dose and *q* dependence (to within uncertainties arising from how available data were collected).

The apparent asymptote of the simulation results to α = 2 at large *q* in Fig. 6 ▸ can be readily understood. In the limit of each pixel or voxel having received a large number of hits *N*, the fractional fluctuations in the number of hits (or blurs) per voxel *N* ^1/2^/*N* will become small, and the blurring of the electron density will become nearly uniform throughout the sample. The uniformity of the blurring will be greater for long-wavelength (large numeric resolution, small *q*) Fourier components of the electron density, since these average over fluctuations in a larger volume. Instead of our elementary damage event corresponding to a local blurring in a small volume of the crystal, in this large-dose, low-resolution limit we can assume a simpler model in which our elementary damage event delivers a uniform dose *D*_0_ that produces a uniform blurring throughout the crystal. After *n* of these events, the total dose received by the sample is *D*_*n*_ = *nD*_0_, and the electron density satisfies ρ(**r**, *D_n_*) = ρ(**r**, *D*_*n*−1_) ⊗ *G*(**r**), where the blur kernel *G*(**r**) is convolved with the real-space density. The Fourier transform of the electron density and thus the diffracted intensity will have the form *I*(*q*, *D_n_*) = *I*(*q*, 0) × [*G*′(*q*)]^2*n*^. Taking *G*(**r**) and thus *G*′(**q**) as isotropic Gaussians, the diffracted intensity can be written as
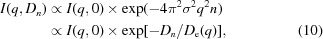
where
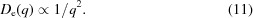
Thus, this simplified model predicts an exponential dependence of intensity on dose and an exponent α = 2 in (4).

Above the protein–solvent glass transition near 200 K, damage processes involving relaxations on large length scales and longer, temperature-dependent timescales may quali­tatively change the evolution of disorder with dose, especially at large doses. Deviations of the local 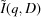, determined by deconvolving the effects of nonuniform irradiation, from the predictions of the simple model used here should provide a useful starting point for the study of these damage processes.

### 4.6. Implications for crystallographic data analysis   

Diffraction scaling programs used in crystallography attempt to correct for changes in Bragg intensities owing to radiation damage. An early approach assumed a linear variation of peak intensity with frame number or dose and used measurements of equivalent peaks in different frames to extrapolate back to the zero-dose intensity. A second approach assumes that the decay of intensities with dose can be described by a linearly increasing *B* factor, *I*(θ, *n*) = *I*_0_[*B*(*n*)sin^2^(θ)/λ^2^], where *B*(*n*) = (1 − *n*)*B*_0_ + *nB*_1_ and *n*, the frame number, is proportional to dose (Otwinowski & Minor, 1997 ▸; Evans, 2006 ▸). This can be rewritten as
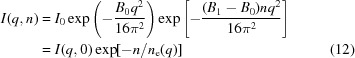
with *n*_e_(*q*) ∝ 1/*q* ^2^. This matches the result of our model and is consistent (within experimental uncertainties) with the local 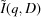 relation we deduced from previous experiments.

This correction can be calculated separately for each set of symmetry-related peaks and their Friedel mates, assuming that all peaks in this set have the same decay rate (Diederichs, 2006 ▸). This requires that the multiplicity of the data set be sufficiently high and that observations of equivalent reflections are well spaced over the data set.

The difficulty in these approaches in accounting for radiation damage is that the measured peak intensities (even when fully recorded) may not have an exponential dependence on frame number (Owen *et al.*, 2014 ▸; Warkentin *et al.*, 2017 ▸), the dose state of the diffracting crystal region does not in general vary linearly with frame number (Warkentin *et al.*, 2017 ▸), and both of these effects are by far the largest for the highest resolution data. Because the crystal is nonuniformly irradiated and nonuniformly damaged, diffraction in any frame reflects a nonlinear weighting of structure factors from crystal regions in various states of decay. Since the local *D*_e_(*q*) ∝ 1/*q* ^2^, the effects of nonuniform irradiation are much more pronounced, and become evident at much smaller exposures, for the highest resolution structure factors. The size, shape and position in reciprocal space of a structure-factor peak evolve with dose, because mosaicity, the spread in lattice constants within the illuminated volume, and the average lattice constant generally increase with dose. Depending on the incident beam divergence and energy spread, the initial crystal mosaicity and its rate of increase with dose, the incident beam fluence or dose per frame and the sample rotation per frame, the resulting intensity variations with exposure time can introduce large errors in nominally fully recorded peak intensities. Recording high-multiplicity data can average over these effects, but there is no reason to expect that the ‘average’ for a set of equivalent reflections, or its extrapolation to frame *n* = 0 based on damage models implemented in current scaling programs, will correspond to the *n* = 0 structure factor, especially in the highest resolution shells. This may contribute to the rapid degradation of *R* factors within each shell as the resolution limit of a data set is approached (Holton *et al.*, 2014 ▸).

The good news, evident from the present work, is that our understanding of radiation damage is improving. It should soon be possible to implement much more sophisticated models, and perhaps also improved crystallographic data-collection protocols that include the measurement of key damage-related parameters, to allow more accurate correction of measured intensities. This could help close the *R*-factor gap between protein and small-molecule structures. Whether the corrections are large enough to significantly impact structural models and mechanistic understanding remains to be determined.

## 5. Conclusions   

We have shown that the experimentally observed diffracted intensity decays and their resolution or *q* dependence, arising from radiation damage to biomolecular crystals, can be explained by assuming a locally exponential relation 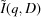 between diffracted intensity and dose with a half-dose *D*_1/2_(*q*) ∝ 1/*q* ^α^ where α ≃ 1.7, and by accounting for the effects of non-uniform irradiation, damage and diffraction during data collection. The very strong dependence of *D*_1/2_(*q*) on *q* increases the effects of both radiation damage and of non­uniform irradiation on measured intensities (and the structure factors derived from them) in the highest resolution shells. Consequently, the 20–30 MGy Henderson or Garman dose limit, which has long been used as a rule of thumb in crystallography, should be replaced with a metric that depends on the initial maximum resolution of a data set, and the application of this metric should account for nonuniform irradiation and diffraction during data collection. Radiation-damage models that have been long used to fit data for *I*(*q*) versus dose implicitly assume uniform sample irradiation and do not apply. Both an exponential dose dependence for 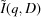 and *D*_1/2_(*q*) ∝ 1/*q* ^α^ with α ≃ 2 follow from perhaps the simplest physically plausible model, in which damage events cause random, local blurring of the electron density. Experimental deviations from these model predictions, especially at temperatures above the protein–solvent glass transition where radiation sensitivity rapidly increases, should guide the development of a more complete model.

## Supplementary Material

Details of the simulations, Supplementary Tables listing experimental and simulation parameters, Supplementary Figures and captions to Supplementary Movies. DOI: 10.1107/S2052252519008777/jt5036sup1.pdf

Supplementary Movie S1. DOI: 10.1107/S2052252519008777/jt5036sup2.mp4

Supplementary Movie S2. DOI: 10.1107/S2052252519008777/jt5036sup3.mp4
