# Supplementary material for: Resolution and dose dependence of radiation damage in biomolecular systems
Source: IUCrJ. 2019 Sep 18;6(Pt 6):1040–53. doi: 10.1107/S2052252519008777 (PMC6830208; doi:10.1107/S2052252519008777)
Supplement: Supplementary file 1 [file m-06-01040-sup1.pdf]

IUCrJ

Volume 6 (2019)

Supporting information for article:

Resolution and dose dependence of radiation damage in biomolecular systems

Hakan Atakisi, Lauren Conger, David W. Moreau and Robert E. Thorne

S1. Fitting of experimental intensity vs resolution and dose data when the crystal orientation was unknown

When the crystal orientation was not given, we assumed that one of the three crystal axes was aligned with the beam direction, and performed three separate fits, assuming alignment of each axis. If the crystal dimensions along the axes were substantially (e.g., an order of magnitude) different, we used the orientation that gave the best fit; the other orientations gave substantially worse fits, regardless of the choice of α. When the crystal dimensions along the three axes were comparable (e.g., within a factor of 2), then the best-fit α would change with assumed orientation by at most ± 0.2, varying with the ratio of the beam FWHM to the crystal size.

S2. Simulations of random blur model for radiation damage

S2.1. 2D simulations

For 2D crystal simulations, we used as a unit cell a grey-scale image with a resolution of 1024 × 512 pixels and with pixel values from 0 to 512 (and a maximum value in the image of ~300). The pixel values correspond to the number of electrons per unit pixel area. Effects of initial static crystal disorder were modeled by randomly rotating the image in each unit cell by ±0.5–±1°.

For each x-ray hit, a small *n*×*n* (typically 5×5) pixel interaction region centered at (x_i, y_j) on the crystal is randomly selected and copied into a matrix. To account for the edges of this region, the matrix is extended to a 3*n*×3*n* matrix by reflecting the original matrix about each edge; e.g., in 1D, an original array "a b c d" becomes "d c b a | a b c d | d c b a". This is the default edge handling method of the *scipy* library of Python. A Gaussian spatial filter is applied to each of the pixels within the *n*×*n* interaction region of the extended matrix. The 2D Gaussian kernel applied to a pixel at (x_i, y_j) is given by

$$G\left(x_{i'} - x_i, y_{j'} - y_j\right) = \frac{1}{2\pi\sigma^2} exp\left(-\frac{\left(x_{i'}-x_i\right)^2+\left(y_{j'}-y_j\right)^2}{2\sigma^2}\right), \quad (8)$$

with σ between 0.5 and 1, the small σ values causing the Gaussian to decay to near zero in only a few pixels. The intensity of a pixel within the interaction region before and after the kernel is applied to that pixel are $r\left(x_i, y_j\right)$ and

$$\rho'\left(x_i, y_j\right) = \sum_{i',j'} G\left(x_{i'} - x_i, y_{i'} - y_i\right)\rho\left(x_{i'}, y_{j'}\right), \quad (9)$$

where the sum is over all pixels in the extended matrix. The pixel value in the original image matrix is updated after the calculation, while the extended matrix values are not changed. Fig. S7 shows the effect of the Gaussian blur.

Simulations were performed using $m \times m$ arrays of unit cells with m ranging between 4 and 128, and using unit cells with grid sizes ranging from 64×32 to 1024×512 pixels. Results with smaller unit cells became largely independent of m for $m \geq 8$. Consequently, $m=32$ was used in most simulations, with $m=16$ used for the largest unit cell. Simulations were run until the random hits caused the diffraction peaks in the highest resolution shell to fall below the background level, which corresponded to roughly 5-10 hits/pixel. Each simulation was divided into 30-50 segments, and after each segment the FFT of the crystal was calculated. The intensity of each diffraction peak is calculated by subtracting the squared local background pixel count from the squared peak count. The diffraction peaks were binned into resolution shells defined by upper and lower resolutions. The integrated intensity of the diffraction peaks, normalized by the undamaged intensity, in each resolution shell is plotted against the average number of hits per pixel.

Fig. 5 illustrates the model using a two-dimensional crystal. Each unit cell contains a grey-scale image of a flea, and the grey-scale value of each pixel corresponds to the electron density there. The Fourier Transform of the initial, undamaged crystal has strong diffraction peaks extending out to the maximum q / resolution of the initial image. After some large number of hits, the real space electron density is blurred throughout the sample, and the Fourier transform decays much more rapidly with q. Movies S1 and S2 show the evolution of the crystal's electron density and the squared amplitude of its FFT, proportional to the diffracted intensity, with dose.

S2.2. 3D simulations

Computationally much more intensive simulations of radiation damage to 3D crystals were performed to assess whether the whether the shapes of the dose curves $I(q,D)$ and the exponent α in Eq. 4 depended on dimension. PDB entry 3E4H, for tetragonal crystals of the 29 residue plant protein Cyclotide varv F at 1.8 Å resolution was chosen for the simulation. The 3D electron density map of the asymmetric unit was calculated from the PDB file (with hetero atoms removed) using *phenix.fmodel*, which created an output in *.mtz* format. This map was discretized using double-precision floating-point format and between 16 and 128 points in each dimension (limited by the available memory for the simulation) by reading from the *.mtz* file using the Phenix tool *phenix.map_value_at_point*, and is shown in Fig. S4. The simulations were performed using the *m2.2xlarge* cluster instance of Cornell Advanced Computing's Red Cloud, which has 28 cores and 192 GB of RAM. This allowed simulation of crystals with 16×16×8 unit cells when using 128^3 voxels per cell; 16×16×16 unit cells were used for all other simulations. The largest simulations took about 7 hours.

Table S1 Experimental parameters for the radiation damage data analysed here.

Reference	Protein	Crystal dimensions (μm) & initial orientation	Beam shape	Beam h × v FWHM (μm)	λ (Å)	Data Collected	Comments	Original dose calculation
Liebschner 2015	Thaumatin	99×145×120<br>c axis aligned with beam	top-hat profile given	51×29	0.979	oscillation<br>2° frames	Only fully recorded reflections used<br>No LP correction	RADDOSE v2<br>no rotation
Sliz 2003	US2	100×100×60<br>orientation not given	gaussian	65×20		oscillation<br>1° frames		Not calculated<br>Fluence given
	HLA	150×150×40<br>orientation not given	gaussian	65×20	1.033	oscillation<br>1° frames	Low and high dose data don't align	Not calculated<br>Fluence given
	λ3	300×300×20<br>orientation not given	gaussian	65×20		oscillation<br>1° frames	Low and high dose data don't align	Not calculated<br>Fluence given
Bourenkov 2009	FAE	size >> beam<br>orientation not given	gaussian	40×30	0.99	rotation<br>70°, 0.25 and 0.5° frames		RADDOSE v1<br>no rotation
	Insulin	35×35×35<br>orientation not given	gaussian	40×30	0.97	rotation<br>300°, 1° frames		RADDOSE v1<br>no rotation
	P19–siRNA-1B	25×25×200<br>orientation not given	gaussian	40×30	0.99	rotation<br>28.8°, 0.8° frames		RADDOSE v1<br>no rotation
Teng 2000	Lysozyme	100×100×40<br>orientation not given	gaussian	295×270	1	oscillation<br>1° frames	Crystal rotated during long exposure, then frame acquired	Manual
Owen 2006	Holoferritin ("holo1")	200×200×200<br>orientation not given	top-hat	100×100	0.939	rotation<br>~30° per set up to 12 sets	Overall $D_{1/2}$ and $D_{1/2}$ for 2 res. shells given for "holo1" crystal	RADDOSE v1<br>no rotation
Warkentin 2010	Thaumatin	size > beam<br>orientation not given	top-hat	100×100	0.984	oscillation<br>5° frames		Manual<br>no rotation

Table S2 Simulation parameters for the radiation damage data analysed here.

Reference	Protein	Crystal shape & dimensions (μm)	Beam shape & dimensions FWHM h × v (μm)	Data simulated	Comments
Liebschner 2015	thaumatin "xtal 01"	rectangular prism 99×145×120 beam along c	rectangular top-hat 51×29	oscillation 2° frames	
			actual beam shape (See Fig. S5)	oscillation 2° frames	
Sliz 2003	US2	rectangular prism 100×100×60 beam along short dim.	h: top-hat v: Gaussian 65×20	oscillation 1° frames	
	HLA	rectangular prism 150×150×40 beam along short dim.	h: top-hat v: Gaussian 65×20	oscillation 1° frames	
	λ3	rectangular prism 300×300×20 beam along short dim.	h: top-hat v: Gaussian 65×20	oscillation 1° frames	
Bourenkov 2009	FAE	rectangular prism 100×100×75 beam along short dim	Gaussian 40×30	rotation 70°, 0.25 and 0.5° frames	Assumed 30 Å for lowest res. No scaling to account for change in illuminated volume applied.
	Insulin	rectangular prism 35×35×35	Gaussian 40×30	rotation 300°, 1° frames	Assumed 40 Å for lowest res. No scaling to account for change in illuminated volume applied.
	P19	rectangular prism 25×25×250 beam ⊥ long dim	Gaussian 40×30	rotation 28.8°, 0.8° frames	Assumed 30 Å for lowest res. No scaling to account for change in illuminated volume applied
Teng 2000	Lysozyme	rectangular prism 100×100×40	Gaussian 295×270	oscillation* 1° frames	*Continuously rotated during long irradiation, before recording diffraction pattern from same 1° wedge
Owen 2006	Holoferritin	rectangular prism 200×200×200	rectangular top-hat 100×100	oscillation 30° frames	Fit parameters adjusted so half doses reported for two resoluton shells are correctly predicted and so that the reported overall intensity versus dose curve is well fit.
Warkentin 2010	Thaumatin	rectangular prism 300×300×300	rectangular top-hat 100×100	oscillation 5° frames	

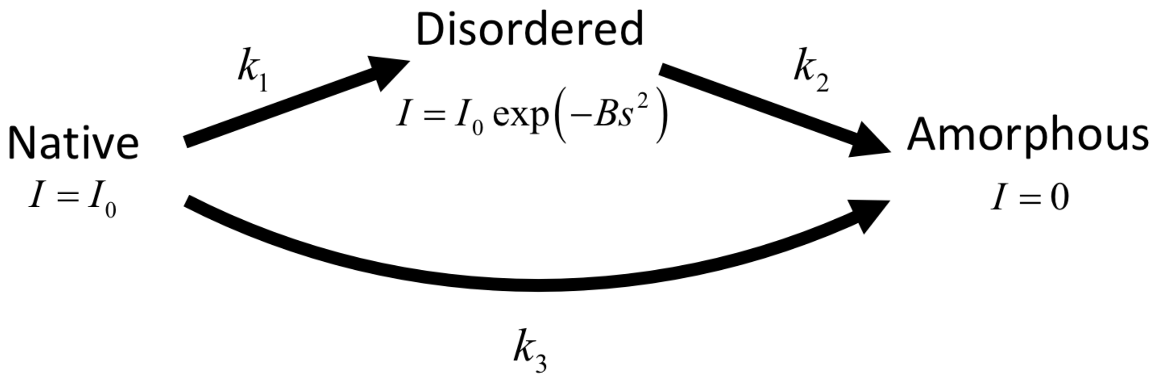

Figure S1 The radiation-damage model of Blake & Phillips as modified by Hendrickson (Blake *et al.*, 1962; Hendrickson, 1976). An initially undamaged crystal region may become either disordered, corresponding to an increased average B factor, or amorphous, corresponding to a complete loss of Bragg scattering. Undamaged crystal becomes disordered with a "rate constant" (fraction per unit dose) k_1 and this disordered crystal becomes fully amorphous with a rate constant k_2. Undamaged crystal can also directly proceed to the amorphous state at rate k_3. Previous fits to experimental data typically found $k_3 \approx 0$ (Hendrickson, 1976; Sliz *et al.*, 2003; Warkentin & Thorne, 2010).

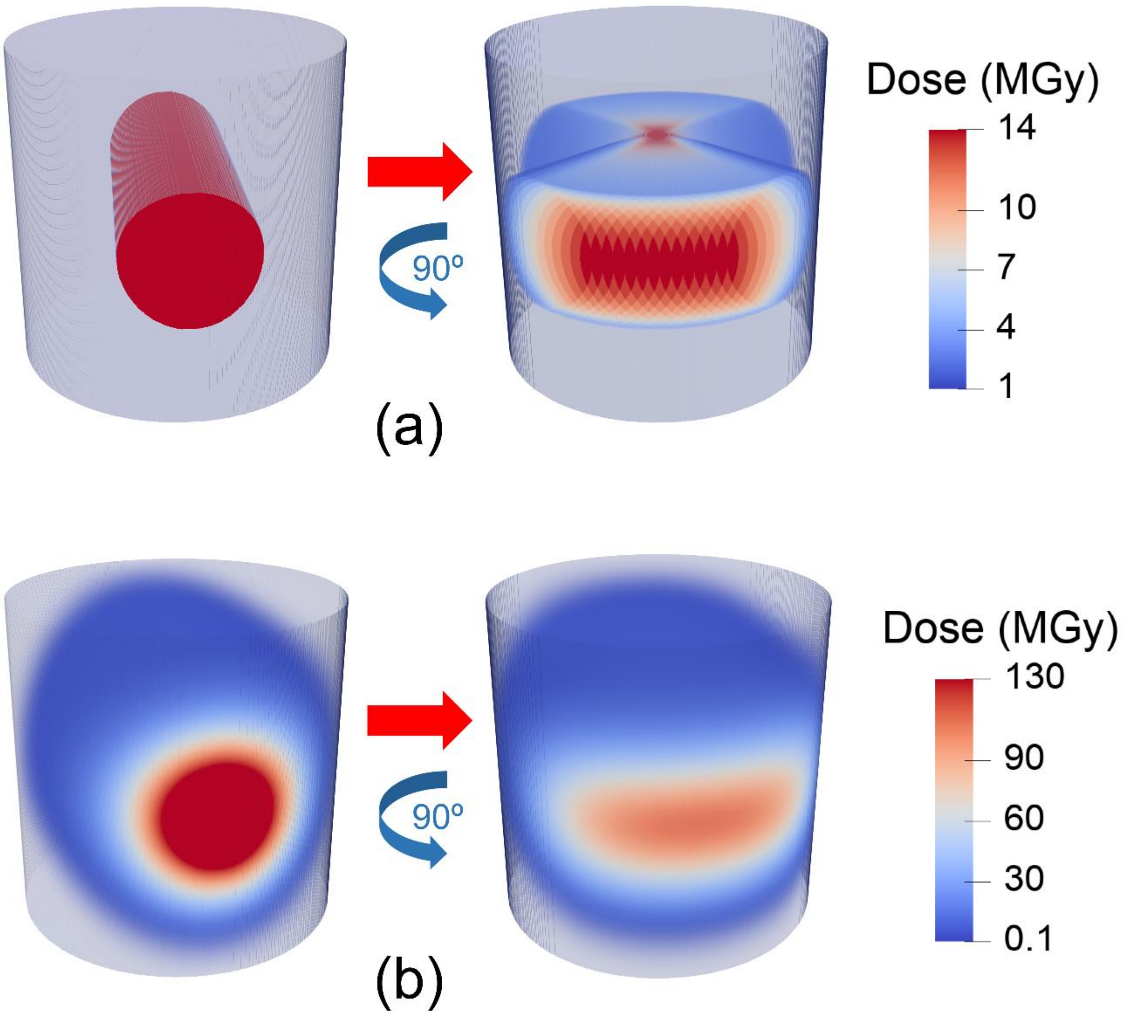

Figure S2 Dose distribution received by a cylindrical crystal illuminated by an x-ray beam with (a) a flat-top flux profile and (b) an uncollimated Gaussian flux profile, when held in a fixed orientation (left) and when rotated by 90°. The crystal diameter and height are 100 μm and the FWHM of the Gaussian and the diameter of the flat-top beam are both 35 μm. Figures are prepared using the software ParaView.

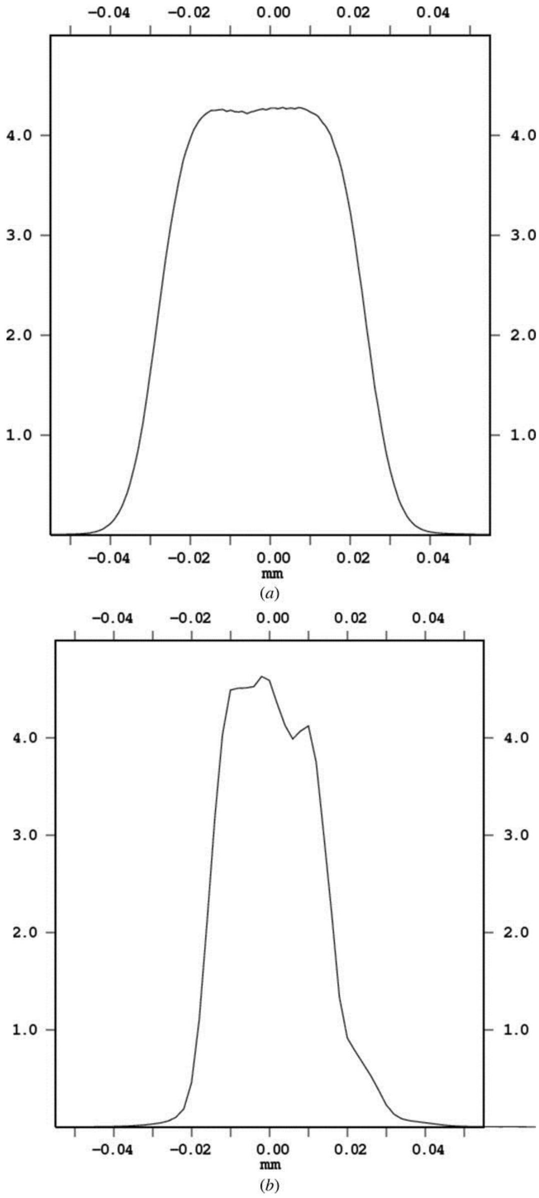

Figure S3 Measured x-ray beam profile in horizontal (a) and vertical (b) reported by Liebschner et al. (Liebschner *et al.*, 2015) in their Figure 1, reproduced here for convenience.

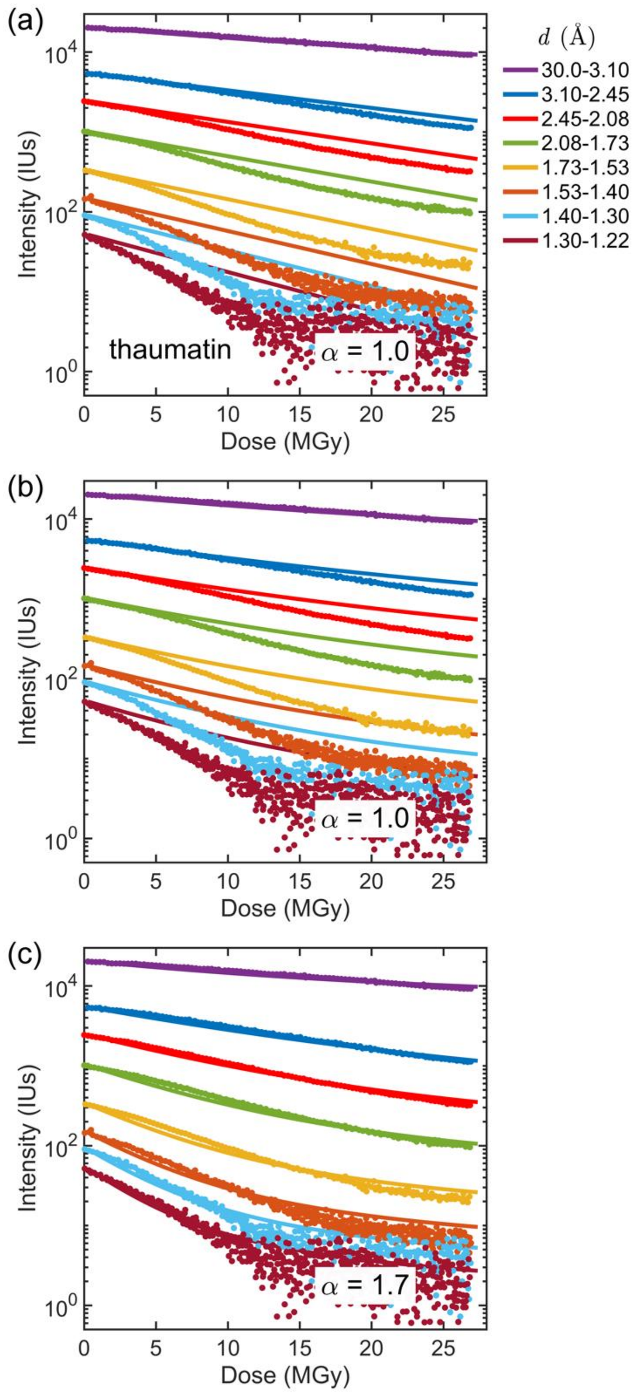

Figure S4 Experimental data (solid circles) for integrated intensity in resolution shells versus dose for thaumatin crystals at 100 K, measured by Liebschner et al. (Fig. 4 in the original manuscript) (Liebschner *et al.*, 2015). Figure 2 shows the same data normalized in each resolution shell by the zero-dose intensity. The solid lines indicate results from simulations assuming (a) a perfect top-hat incident x-ray beam profile and an exponent $\alpha = 1$ in Eq. 4; (b) the measured beam profile (Fig. 1 in the original manuscript, reproduced in Fig. S3) and $\alpha = 1$; and (c) the measured beam profile and a best-fit exponent $\alpha = 1.7$.

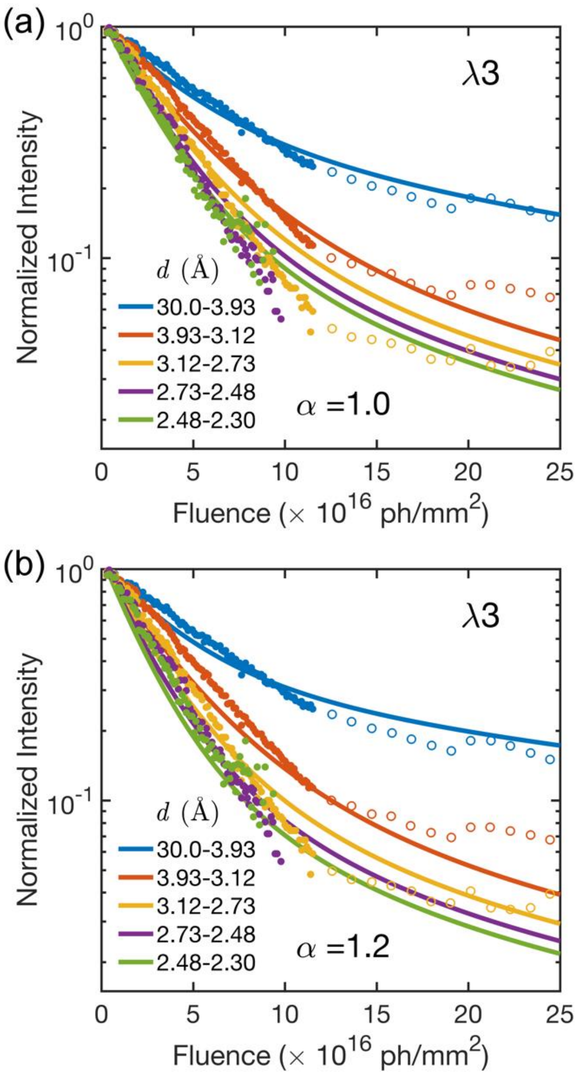

Figure S5 Experimental data in the low dose (solid circles) and high dose (open circles) regions for integrated intensity in resolution shells versus incident fluence (in ph/mm^2, proportional to dose) reported by Sliz *et al*. (Sliz *et al.*, 2003) (Fig. 1 in the original manuscript) for a crystals of $\lambda 3$ at 100 K. The solid lines indicate results from simulations assuming a top-hat incident beam profile in the horizontal direction and a Gaussian profile in the vertical direction (based on descriptions of the experimental setup), with (a) $\alpha = 1$ and (b) a "best-fit" value chosen based on visual comparison. The discontinuity in slope between the low and high dose data suggests issues in data collection or analysis.

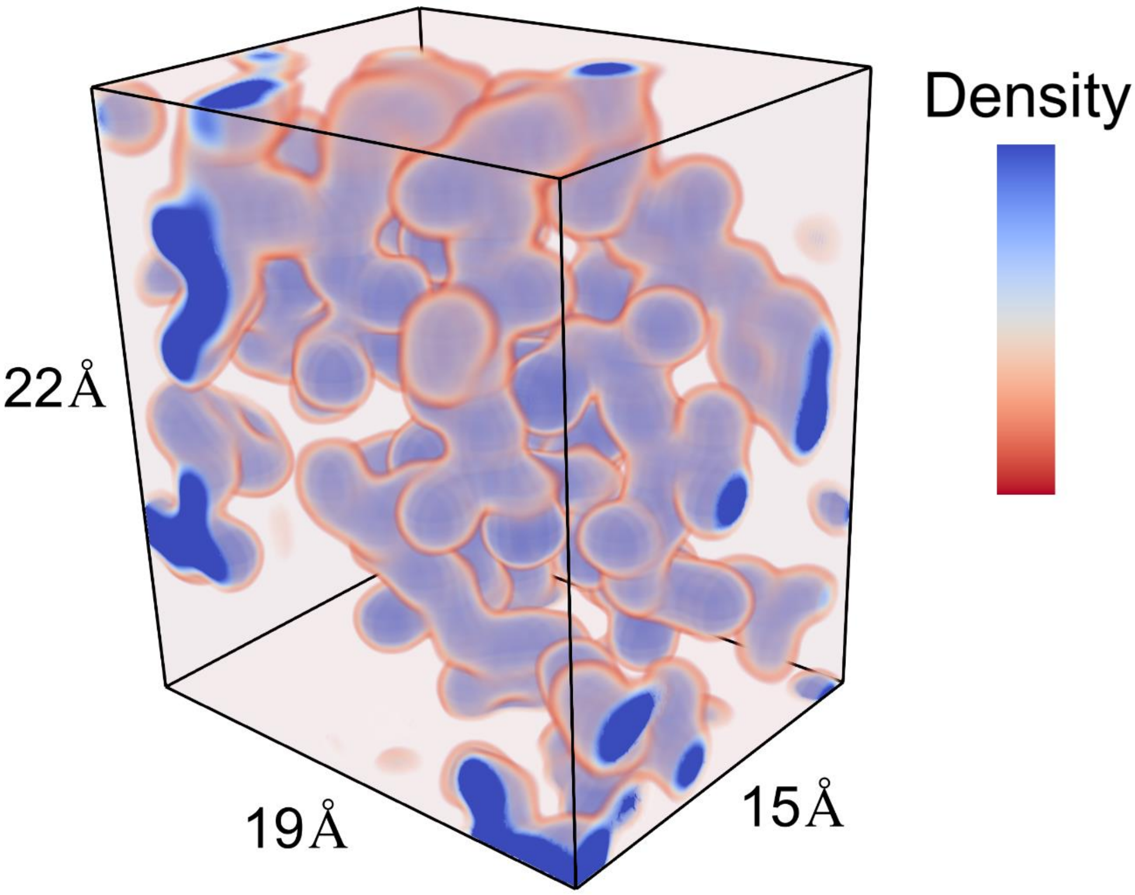

Figure S6 Discretized electron density map based on PDB entry 3E4H using 128 grid points in each dimension. Shown is the smallest rectangular prism enclosing the protein chain in the asymmetric unit. This was used as the unit cell for simulations. The actual crystal of 3E4H has a cubic unit cell of size 82 Å and contains 48 copies of the ASU, and was too large for our simulations.

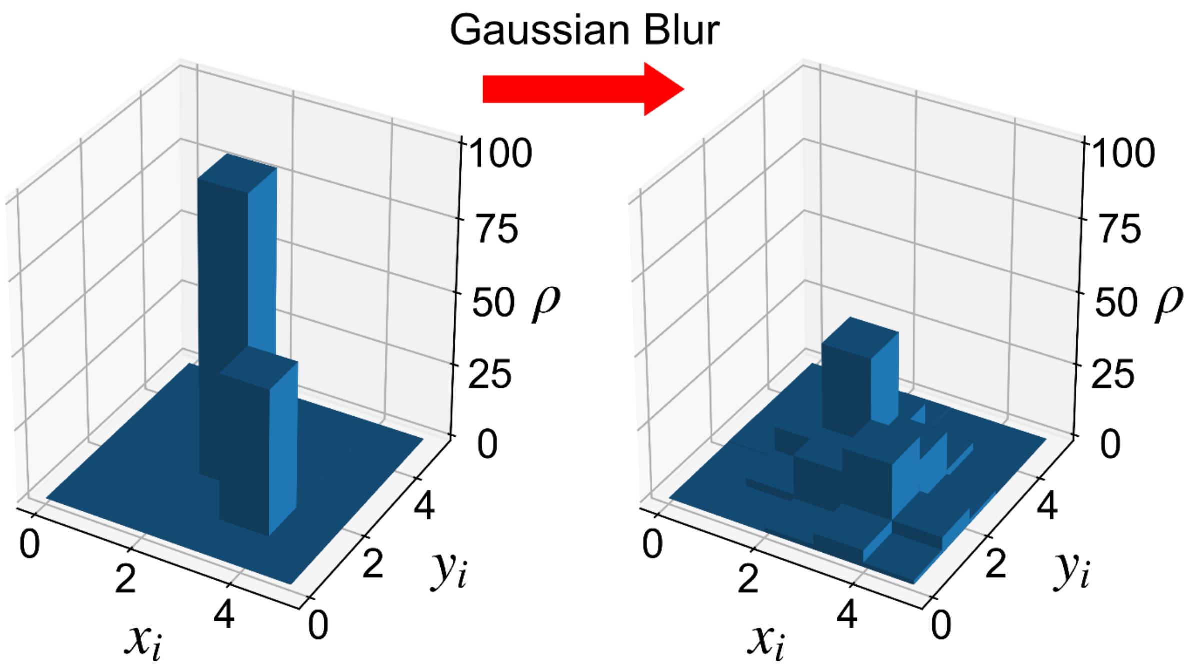

Figure S7 Gaussian blur with σ=0.6 applied to a 5×5 pixel region, with edge handling performed as described in Section S1. Pixel counts correspond to electrons/pixel or electron density. The sum of pixel counts - corresponding to the total number of electrons - is conserved in the blurring. The Gaussian function vanishes beyond ±3σ from the central pixel.

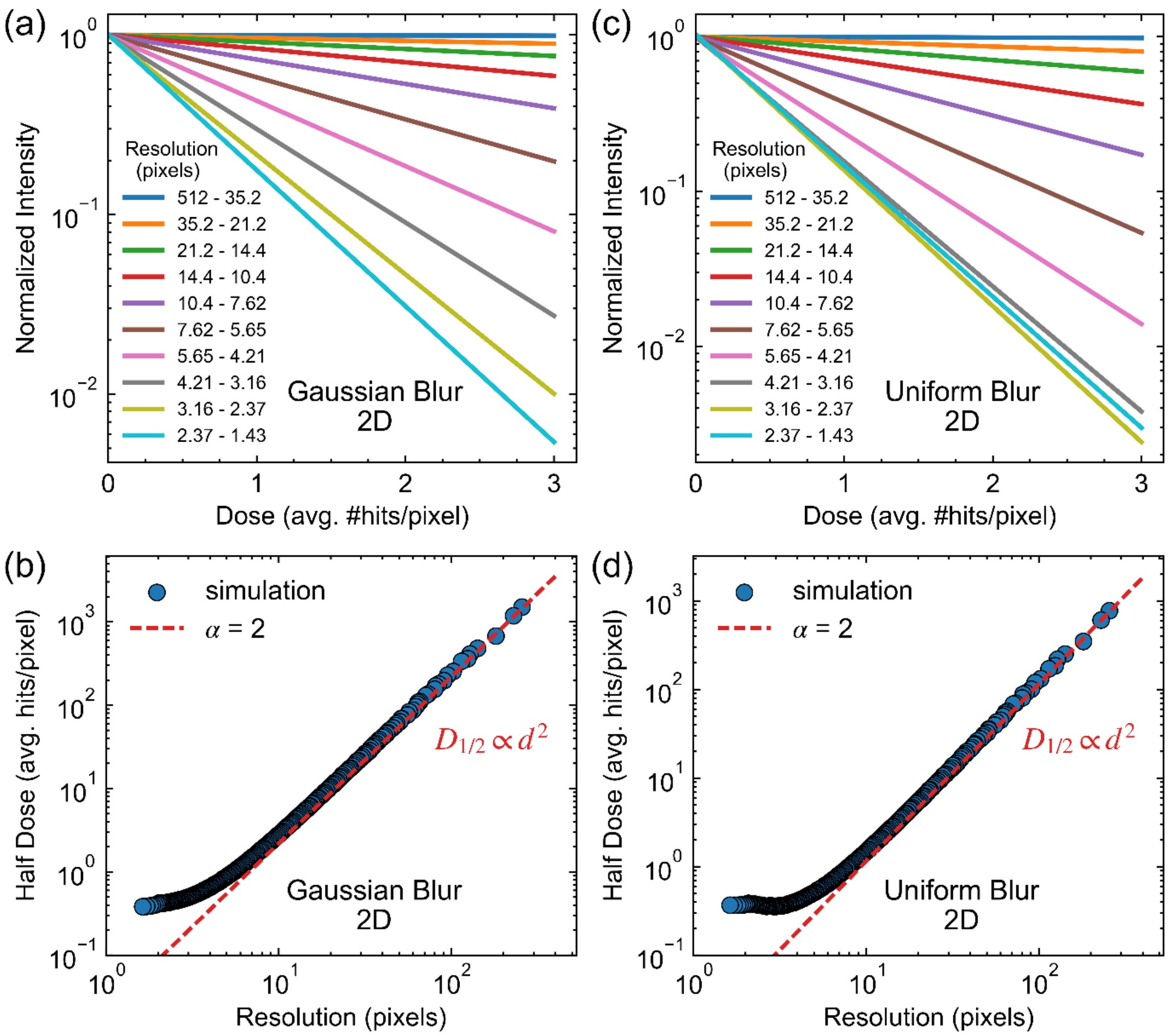

Figure S8 Results of 2D simulations of radiation damage as described in Section S1 using (a),(b) Gaussian and (c),(d) uniform blur kernels, for a uniformly irradiated sample. (a) and (c) show normalized integrated intensity in resolution shells versus dose. (b) and (d) show half dose $D_{1/2}(d)$ in a resolution shell vs resolution *d*. Both kernels yield purely exponential intensity decays with dose in each resolution shell, and the same exponent α (within errors) for $D_{1/2}(d) \propto d^{\alpha}$.

Movie Captions

Movie S1 Video showing the evolution of the electron density of the 2D crystal in Fig. 5 as the number of "hits" increases, generated using our model of radiation damage as a series of Gaussian blurs applied at random locations. Audio as in Video S2.

Movie S2 The evolution of the square of the amplitude of the FFT of the electron density shown in Video S1, corresponding to the evolution of the crystal's diffraction pattern. The audio was generated by mapping the magnitude of the q vector of each FFT peak onto a frequency between 50 Hz (for q=0) and 20 kHz, and generating tones for each with an amplitude proportional to the square of the FFT peak amplitude. The large q peaks / high frequency tones disappear rapidly, while the small q peaks / low frequency tones fade out very slowly.

References

Blake, C., Phillips, D., CCF, B. & DC, P. (1962). *Biological Effects of Ionizing Radiation at the Molecular Level*, Vol. pp. 183–191. Vienna: International Atomic Energy Agency.

Hendrickson, W. A. (1976). *J. Mol. Biol.* **106**, 889–893.

Liebschner, D., Rosenbaum, G., Dauter, M. & Dauter, Z. (2015). *Acta Cryst. D*. **71**, 772–778.

Sliz, P., Harrison, S. C. & Rosenbaum, G. (2003). *Structure*. **11**, 13–19.

Warkentin, M. & Thorne, R. E. (2010). *Acta Cryst. D*. **66**, 1092–1100.
